# Glacier algae foster ice-albedo feedback in the European Alps

**DOI:** 10.1038/s41598-020-61762-0

**Published:** 2020-03-16

**Authors:** B. Di Mauro, R. Garzonio, G. Baccolo, A. Franzetti, F. Pittino, B. Leoni, D. Remias, R. Colombo, M. Rossini

**Affiliations:** 1 Earth and Environmental Sciences Department, University of Milano-Bicocca, 20126 Milan, Italy; 2 National Institute of Nuclear Physics (INFN), Section of Milano-Bicocca, Milan, Italy; 3 University of Applied Sciences, Campus Wels, Stelzhamerstr. 23, A-4600 Wels, Austria

**Keywords:** Cryospheric science, Microbial ecology

## Abstract

The melting of glaciers and ice sheets is nowadays considered a symbol of climate change. Many complex mechanisms are involved in the melting of ice, and, among these processes, surface darkening due to organic material on bare ice has recently received attention from the scientific community. The presence of microbes on glaciers has been shown to decrease the albedo of ice and promote melting. Despite several studies from the Himalaya, Greenland, Andes, and Alaska, no quantitative studies have yet been conducted in the European Alps. In this paper, we made use of DNA sequencing, microscopy and field spectroscopy to describe the nature of glacier algae found at a glacier (Vadret da Morteratsch) of the European Alps and to evaluate their effect on the ice-albedo feedback. Among different algal species identified in the samples, we found a remarkable abundance of *Ancylonema nordenskioeldii*, a species that has never previously been quantitatively documented in the Alps and that dominates algal blooms on the Greenland Ice Sheet. Our results show that, at the end of the ablation season, the concentration of *Ancylonema nordenskioeldii* on the glacier surface is higher than that of other algal species (i.e. *Mesotaenium berggrenii*). Using field spectroscopy data, we identified a significant correlation between a reflectance ratio (750 nm/650 nm) and the algae concentration. This reflectance ratio could be useful for future mapping of glacier algae from remote sensing data exploiting band 6 (740 nm) and band 4 (665 nm) of the MultiSpectral Instrument (MSI) on board Sentinel-2 satellite. Here we show that the biological darkening of glaciers (i.e. the bioalbedo feedback) is also occurring in the European Alps, and thus it is a global process that must be taken into account when considering the positive feedback mechanisms related to glacier melting.

## Introduction

Glaciers and ice sheets are not lifeless^1^. It has been demonstrated that several species of microorganisms, algae and small arthropods find their optimal environment on melting ice and snow. These organisms are also able to shape their environment, through a feedback cycle that involves the albedo. In fact, since these organisms are darker than snow and ice, their presence increases the light absorption and promotes the melting of underlying snow or ice^2–4^. On the surface of glaciers, such extremophiles often aggregate with inorganic material (i.e. mineral dust) to form cryoconite^5,6^. Cryoconite is a dark sediment^5,7^ that increases the absorption of visible radiation^4,8^, and promotes the formation of characteristic cryoconite holes^9^ which are globally recognized as an hot spot of biodiversity on ice^10^. Besides living organisms, cryoconite is known to concentrate pollutants such as contaminants and radionuclides^11,12^. This may represent a problem in the future, with a possible secondary release of these substances to the environment^13,14^.

Cryoconite accumulated in cryoconite holes has a limited impact on glacier and ice sheet surface mass balance. Instead, the presence of distributed organic material on the margin of ice sheets recently motivated the formalization of the concept of “bioalbedo feedback”^15–17^. This feedback is associated with the spatial distribution of organic material that increases the absorption of light, promotes the phase transition of ice, produces a film of meltwater on snow- and ice-fields, and allows the growth of the algal population^18,19^. The decrease of ice albedo has important consequences on glacier mass balance^20,21^ and represents an active field of research^22,23^. Recent literature focused on the impact of algae on snow and ice in the Greenland Ice Sheet^24–26^, Iceland^27^, Norway^28^, Himalaya^3,17^, Alaska^29,30^, Sierra Nevada^31^, Andes^32 and Antarctica33^. These studies highlighted the presence of different species of algae on snow and ice, and their role in the albedo decrease. While recent literature focusing on the southwest margin of the Greenland Ice Sheet identified a strong impact of glacier algae on the optical properties of ice^18,24,26,34,35^, this phenomenon remains largely unexplored in the European Alps.

In the Alps, most of the studies deal with the characterization of communities living in melting snow^36^. In contrast, studies of glacier surfaces (i.e. bare ice after snow melting) are sparse. Recently, the glacier alga *Mesotaenium berggrenii* was described in Austrian glaciers^37,38^. Kol^39^ first reports about the filamentous alga *Ancylonema nordenskioeldii* in a glacier close to the Mont Blanc. The latter species is well known from polar icefields^18,24,40^, but its presence in Europe was questionable^41^.

The impact of glacier algae on ice albedo can be studied through spectroscopy data collected both in the field^24^ and from aerial^26^ and satellite sensors^25^. While field spectroscopy data are fundamental for assessing the local impact of glacier algae on the optical properties of ice^24^, remotely sensed data can provide a synoptic view of the phenomenon. In particular, the launch of new satellite missions such as Sentinel-2 and Sentinel-3, from the European Space Agency (ESA) Copernicus program, has created new opportunities for the study of the cryosphere from space^42,43^. The spatial, spectral and temporal resolution of these missions allows the monitoring of changes in both alpine and polar glaciers. Sentinel-2 is particularly suited for mapping spatial and temporal variability of the cryosphere at fine scale^44^, while Sentinel-3 allows a broader perspective^43^. Some studies have already exploited these data for mapping algae distribution in Maritime Antarctica^33^ and Southwest Greenland^25,35^.

The objectives of this paper are to identify the algae living on the surface of an Alpine glacier, and to determine their impact on the optical properties of ice. We addressed these objectives with data collected during a survey at the Vadret da Morteratsch Glacier in the Swiss Alps. This glacier has been the focus of numerous studies in recent decades^4,21,45,46^. In particular, Oerlemans *et al*.^21^ identified a decreasing trend in the albedo of bare ice and attributed it to the accumulation of dust from lateral moraines. More recently, Di Mauro *et al*.^4^ demonstrated that the presence of high concentrations of elemental and organic carbon may have contributed to the albedo decrease on this glacier. Here, we report the identification of glacier algae through DNA sequencing of samples collected on the Morteratsch Glacier at the end of the ablation season on September 2016. Furthermore, we measured the impact of glacier algae on the optical properties of ice using near surface reflectance measurements collected with a field spectrometer, and we identified a reflectance ratio that was correlated with algae density. The spectral measurements were then resampled at the same spectral resolution as the ESA MultiSpectral Instrument (MSI) onboard Sentinel-2 to evaluate the potential of global satellite mission observations for mapping the spatial and temporal distribution of algae in the alpine environment.

## Results and Discussion

### Population densities

**Figure 1:**
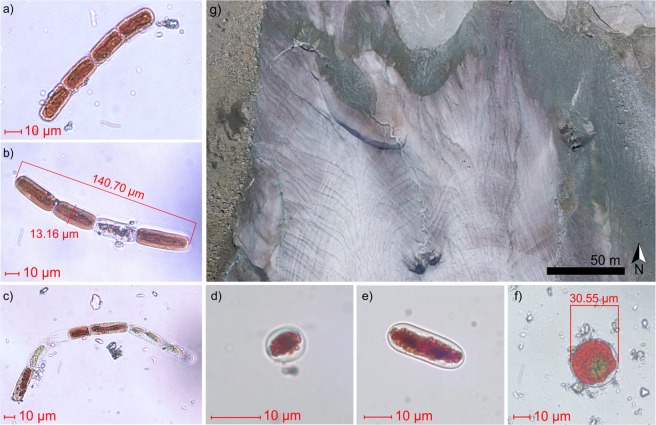
(**a–f**) Light micrographs of algae found at the glacier. (**a–c**) Filaments of *Ancylonema nordenskioeldii*. (**d,e**) unicellular *Mesotaenium berggrenii*. (**f**) red cyst of putative *Sanguina nivaloides* (**g**) Aerial view of the Morteratsch glacier tongue acquired in September 2016 from an Unmanned Aerial Vehicle (UAV)^49^.

Among the algal species identified on the ablation zone of the Vadret da Morteratsch by light microscopy (Fig. 1g), *Ancylonema nordenskioeldii, Mesotaenium berggrenii* and *Sanguina nivaloides* were the most representative. We point out the rediscovery of *A. nordenskioeldii* (Fig. 1a–c) in the European Alps, with a mean density of 2.4 × 10^4^ cells ml^−1^. *A. nordenskioeldii* features a different size (cell length = 30.7 ± 5.7 µm and cell width = 12.7 µm ± 1.0 µm) from those of the polar regions, whose cells have smaller lengths^40,47^, and from the Chilean ones, which feature larger lengths^32^. *M. berggrenii* was present with two distinct non-filamentous varieties (Fig. 1d,e): the *alaskana* variety^48^, easily distinguishable by a single chloroplast characterizing cells after division, was the most widespread species on the glacier with average density of 6.7 × 10^4^ cells ml^−1^. For both glacier algae, only vegetative cells were present, and no zygotes were observed. Red spherical cysts resembling *S. nivaloides* (Fig. 1f) were found in 6 out of 18 samples, with an average density of 767 cells ml^−1^.

The average algal cell density found in this work (i.e. 20.3 × 10^4^ cells ml^−1^) is comparable with those found in South-West Greenland^24,50^. Thus, we might expect that the algae density observed on Vadret da Morteratsch Glacier will have an effect on optical properties similar to that already observed on polar glaciers^24,35^.

### DNA sequencing

**Figure 2:**
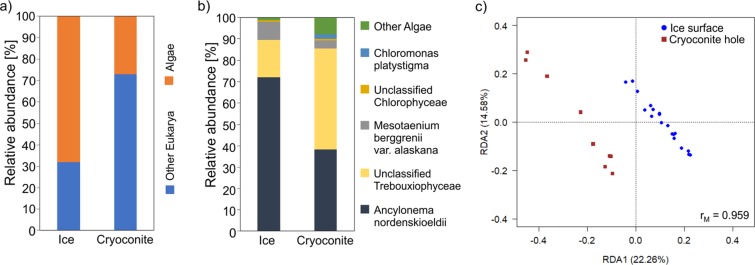
(**a**) Relative operational taxonomic units (OTUs) abundance of algae and other Eukarya in both ice surface and cryoconite holes samples. (**b**) Relative abundance of the algal OTUs grouped in taxa (taxa whose abundance was lower than 1% were grouped in “Other Algae”) (**c**) Biplot from RDA on Hellinger-transformed algal OTU abundance. Each point represents one sample. The analysis includes ice surface samples (blue dots) and cryoconite hole samples (brown squares).

The full classification and the relative abundance of algal Operational Taxonomic Units (OTUs) are reported in Table S1. Results show that algae were relatively more abundant in surface ice than in cryoconite samples (Fig. 2a). Moreover, the composition of glacier algal communities was different from those in cryoconite holes. Particularly, algal communities in ice were dominated by *Ancylonema nordenskioeldii* with an average abundance of 70%, whereas in cryoconite holes members of Trebouxiophyceae were the dominant taxa (Fig. 2b). Redundancy analysis (RDA) showed that algal community structure varied significantly according to the type of sample (cryoconite holes or ice surface) (Table S2).

Indeed, the RDA plot (Fig. 2c) shows that samples cluster according to the supraglacial habitats. Figure 2c also shows that the cryoconite and ice samples are distributed along two parallel directions. This suggests that a factor not investigated in this study (e. g. irradiation, pH, total organic carbon, nutrients concentration) may describe part of the unexplained variance of the algal community^51^. Given the complexity of the supraglacial environment, even the relatively low explained variance (i.e. 36.8%) can be considered satisfactory^52^. We remark that RDA analysis was conducted using only the variable “supraglacial habitats” (i.e. ice surface or cryoconite hole).

Generalized linear models (GLMs) performed on the three most abundant algae showed that the abundance of *Ancylonema nordenskioeldii*, *Mesotaenium berggrenii var. alaskana* and the Unclassified Trebouxiophyceae changed according to the type of sample. In particular, *Ancylonema nordenskioeldii* (Fig. S2) and *Mesotaenium berggrenii var. alaskana* (Fig. S2) were more abundant in surface ice than in cryoconite holes (F_1,26_ > 7.08; PFDR < 0.006), the opposite occurred for the algae belonging to the Trebouxiophyceae (Fig. S3) (F_1,26_ = 21.66; PFDR < 0.006). The taxonomic affiliation of the most abundant OTUs is reported in Table S3.

The results show that most of the eukaryotes living on bare ice are algae, while in cryoconite holes the eukaryotic community is more heterogeneous. One possible explanation could be that glacier algae are well adapted to the surface ice environment and thus able to develop blooms during the melting period. For example, algae such as *Ancylonema nordenskioeldii* and *Mesotaenium berggrenii var. alaskana* produce dark phenolic pigments to protect them from the high solar radiation^10^. This is consistent with the fact that these species are more abundant on the bare ice than Trebouxiophyceae which lack these secondary pigments^53,54^. The dark secondary pigmentation of *Ancylonema nordenskioeldii* and *Mesotaenium berggrenii var. alaskana* may explain the variation in ice reflectance^47^, which will be discussed in the next section.

### Impact of glacier algae on the optical properties of ice

**Figure 3:**
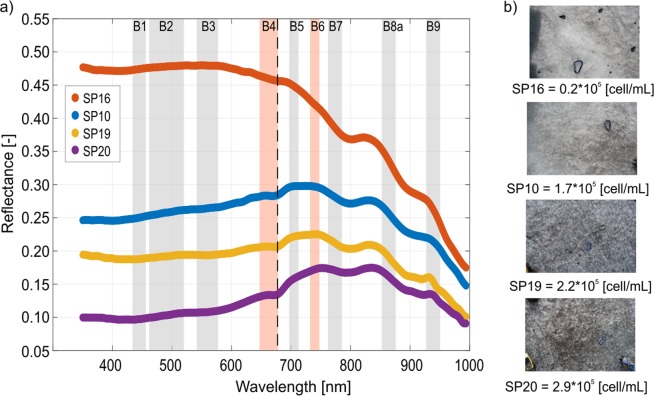
(**a**) Spectral reflectance of bare ice containing different algal densities. The shaded areas represent Sentinel-2 spectral bands (B1–B9). Red areas are the Sentinel-2 bands (B4 and B6) used for calculating the reflectance ratio here proposed. Dotted vertical line indicates the position of the Chlorophyll-a absorption feature at 680 nm. (**b**) Images of the four sampling sites represented in (**a**). Carabiner (length = 7 cm) for scale.

The presence of algae on bare ice causes a decrease of the reflectance at wavelengths shorter than 750 nm because of the absorptions by several intracellular pigments (e.g. Chlorophyll-a, Chlorophyll-b, photosynthetic carotenoids, photoprotective secondary carotenoids, phenols etc.)^15,55^ (Fig. 3a). As previously showed for Greenland^24^, different absorption features can be recognized in the spectra of bare ice containing algae. In particular, an absorption located at 680 nm is usually linked to the presence of Chlorophyll-a. This feature was observed also in the Alaska’s Harding Icefield^29,30^ and in the Yosemite National Park^31^. The specific photosynthetic absorption of algae in the visible wavelengths has been exploited for their estimation from satellite remote sensing data^25,29,30^. In Fig. 3a we show the reflectance spectra of surface ice samples with varying algal abundance. The total concentration of algae spanned from 0.2 × 10^5^ cell/mL (sample ID: SP16) to 2.9 × 10^5^ cell/mL (sample ID: SP20). Photographs of the area measured with the field spectrometer and then sampled for the analysis are showed in Fig. 3b.

**Figure 4:**
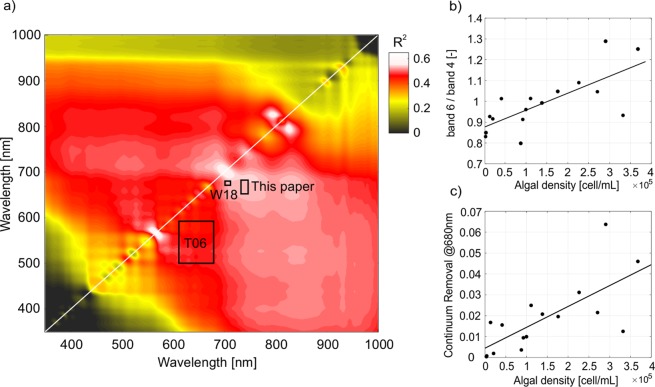
(**a**) Correlation matrix of the coefficient of determination R^2^ created from all possible reflectance ratios using the ASD field spectrometer. Rectangles represents the wavelengths used in previous studies (Takeuchi *et al*.^30^: T06, and Wang *et al*.^25^: W18). (**b**) Linear regression (R^2^ = 0.53, p-value = 0.001) between the reflectance ratio calculated resampling ASD reflectance on Sentinel 2 bands 4 and 6 and the concentration of algae [cell/mL]. (**c**) Linear regression (R^2^ = 0.52, p-value = 0.001) between the continuum removal at 680 nm and the concentration of algae [cell/mL].

A variable selection approach was developed to identify the reflectance ratio index that correlated the most with algae concentration. A hot spot of correlation between spectral ratios and concentration of algae was found at wavelengths between 600 and 700 nm. In particular, the red-edge ratio between reflectance at 750 nm and 650 nm resulted in the highest correlation coefficient (R^2^~0.6). We compared our correlation hot spot with other indices proposed by Takeuchi *et al*.^30^ (T06 in Fig. 4a) and Wang *et al*.^25^ (W18 in Fig. 4a). The correlation coefficient for these two indices was respectively 0.4 and 0.5. However, these indices were developed for tracking variations in algal abundance from satellite platforms, thus using sensors characterized by a different spectral resolution. In particular, W18 index was calculated using the relatively high spectral resolution of the Ocean and Land Colour Instrument (OLCI) sensor on board the Sentinel-3 platform. Despite the relatively high correlation found between W18 and algal density, the use of Sentinel-3 for mapping the algae spatial and temporal distribution in alpine areas is hampered by its too coarse spatial resolution (300 m pixel size). Conversely, the use of the Sentinel-2 satellite characterized by a high spatial resolution (10 to 20 m pixel size) has proved successful for studying alpine glaciers^44,56^.

Reflectance measurements collected with the field spectrometer were resampled (i.e. averaged) using the bandwidth of the MSI on board the Sentinel-2 platform. The reflectance ratio that best correlated with the algal density (R^2^ = 0.53, p-value = 0.001) was the one corresponding to Sentinel-2 band 6 (centered at 740 nm) and band 4 (centered at 665 nm) (Fig. 4b). No significant relation was found between the 740/665 nm ratio and the inorganic sediments found in surface ice (R^2^ = 0.07, p-value >0.05). This result fosters the use of this reflectance ratio for mapping glacier algae. Reflectance ratios of wavelengths across the red edge position are established methods for mapping autotrophic life from satellite data^57^. We here propose the use of the spectral ratio between the reflectance at 740 nm and 665 nm as a useful tool for mapping the presence of algae on ice using remote sensing data collected by different platforms (e.g. satellite sensors, airborne sensors and unmanned aerial vehicles). The relatively weak correlation found between the reflectance ratio and the algae concentration (Fig. 4b) may be explained by other variables related to glacier ice that are not considered in this paper, such as the presence of mineral dust, soot, melt water, grain size, ice density etc. In particular, the reflectance ratio proposed in this paper shows a higher scattering for algal densities greater than 3 × 10^5^ cell/mL. This represents a source of uncertainty in using the reflectance ratio to estimate algal abundance over wider areas using remote sensing. Further research is needed to validate the application of this method at alpine or polar scales.

We exploited an additional approach to evaluate the correlation between the reflectance spectra and the algal abundance, i.e. the continuum removal applied on the Chlorophyll-a absorption feature at 680 nm. We found a significant correlation (R^2^ = 0.52, p-value = 0.001) between the continuum removal and the concentration of algae in surface ice samples. The absorption feature of Chlorophyll-a due to algae is very narrow (Fig. 3a) and can be resolved only from hyperspectral data. Thus, this index can be proposed for mapping algae from high spectral resolution unmanned aerial systems^58^ and hyperspectral satellite data^4,59^. In contrast, the reflectance ratio that we propose in this paper requires less sophisticated measurements to be calculated. While for the Greenland Ice Sheet, it has been demonstrated that the biological darkening of ice is more important than the inorganic one^24^, this may not hold true for nonpolar glaciers, where the availability of mineral impurities from the proglacial area and from melting ice can induce a stronger albedo feedback. The decoupling of the impact of organic and inorganic particles on the optical properties of ice is still unresolved. In this context, the integration of hyperspectral imaging data with radiative transfer modeling could be a promising tool for studying the bioalbedo feedback at different scales.

## Conclusions

In this paper, we have reported the occurrence of glacier algae at a glacier (Vadret da Morteratsch, Switzerland) of the European Alps. In our samples, we found an average algal concentration of 14.2 × 10^4^ cells ml^−1^, which is comparable with sites on the Greenland Ice sheet. The characterization of the communities of both cryoconite holes and surface ice samples showed that cryoconite holes host a more diverse eukaryotic community including heterotrophs, while bare ice is mostly dominated by the autotrophic algae. This may be due to the algae’s capability in colonizing such a harsh and virtually competition-free environment, while cryoconite holes provide more shelter against abiotic stress. In particular, we documented the presence of *Ancylonema nordenskioeldii*, a glacier algal species that has never previously been quantitatively documented in the Alps, and that is known to dominate algal blooms in polar regions. We also report the effect of this bloom on the optical properties of bare ice during the ablation season. Typical absorption features of photosynthetic pigments were detected in reflectance spectra. The reflectance ratio between 740 nm and 665 nm was the index that best correlated with algal abundance and not with inorganic sediments. This index appears promising for the estimation of the abundance of algae on glaciers from remotely sensed data such as those from the ESA Sentinel-2 satellite mission. However, for a wider application of the proposed approach, further validation datasets are needed.

The presence of algae on glacier ice increases the absorption of solar radiation, fostering the ice-albedo feedback during the melting season. Our dataset represents the first direct evidence of the impact of glacier algae on the optical properties of ice in the European Alps, and it is intended to pave the way for future studies on the bioalbedo feedback in the Alps. The identification of all the players involved in ice darkening is a fundamental task for understanding surface glacier melt, and for predicting the response of Alpine glaciers to future climate change.

## Materials and method

### Field spectroscopy and sampling

On September 13^th^ 2016, a campaign was conducted on the ablation zone of the Vadret da Morteratsch Glacier (46°24′34″N, 9°55′54″E), an alpine valley glacier located in the Bernina Massif (4049 m a.s.l, Raethian Alps, Switzerland-Italy). Morteratsch is a large glacier (area ~7.5 km^2^) in the Bernina range, with an altitudinal range of ~2000 m. The glacier snout is located at 2100 m a.s.l. The glacier is characterized by a continental climate, with ablation seasons that can last up to three months during warm summers. This glacier has been extensively studied in recent years^4,21,45,46^. Morteratsch Glacier has been rapidly retreating^21^, and it represents the perfect test bed for studying the impact of glacier algae in the European Alps.

During the campaign, field spectroscopy data were measured with a HandHeld Analytical Spectral Devices (ASD) Field Spec (spectral range = 325–1075 nm, spectral sampling interval = 1 nm). The hemispherical conical reflectance factor (HCRF) was calculated by normalizing the reflected radiance with the incident radiance measured from a calibrated Spectralon© panel. Each acquisition was the average value of 15 spectra. A levelled bare optical fiber (field of view = 25°) was used to collect data at 80 cm from the ice surface (footprint diameter = 35 cm). All measurements were collected around midday for minimizing the effect of the changing solar illumination. Further details on the methodology can be found in previous papers^4,60^. Spectral reflectance was acquired at 18 sampling points distributed on bare ice in the ablation zone of the glacier. For each sampling point, we collected surface material in correspondence of the spectrometer field of view. Since a strong variability of ice properties can be found on the ablation area of Alpine glaciers, the sampling methodology represents a complexity in spectroscopy of ice. For this reason, we paid much attention in sampling ice exactly from the area measured by the ASD field spectrometer.

From reflectance data, we calculated all possible spectral ratios and we created a series (n = 423801) of linear regression models between different spectral indexes and the algal density. This variable selection analysis has been already used for other type of impurities^60^, and it is useful for identifying hot spot of correlation in spectral data. We resampled (i.e. averaged) the reflectance measured with the ASD spectrometer to the spectral resolution of Sentinel-2. This sensor features a high spatial resolution (up to 10 m in the visible spectrum)^61^ and it is promising for mapping glacier algae distribution from space^33^.

Furthermore, we calculated the continuum removal (between 655 and 700 nm) at the Chlorophyll-a absorption feature located at 680 nm^31^. The continuum removal quantifies the absorption features at specific wavelengths, normalizing the reflectance spectra to a common baseline^62^. This is achieved by approximating the continuum between local spectral maxima through straight-line segments: a value of 1 is assigned to the local maxima, and a value between 0 and 1 is obtained in correspondence of the absorption features. The continuum removal calculated at 680 nm was then directly compared with algal concentration through linear regression analysis.

### Cell counting

Eighteen samples of ice were placed inside 50 ml conical bottomed phials. These were kept in freezing condition and transported to Milano-Bicocca University, where they were preserved at −30 °C. For the laboratory analysis, the samples were completely thawed at room temperature. The algal classification by light microscopy and count were performed on fresh material. Identification keys^63^ and literature^24,36,38,64,65^ were used to identify the algae. The organisms were counted by inverted microscopy (400× magnification) and a camera, pictures were analyzed using AxioVision software, density and biovolume were estimated as reported in literature^66^. In order to characterize the size of the identified organisms, we measured the length and width of at least 50 cells for each species^67^. Using a Whatman GF/C (1.2 µm) glass fiber filter we also quantified the total inorganic sediments contained in samples collected on the Morteratsch Glacier.

### DNA extraction and sequencing

Ice was melted and centrifugated at 12000 × g for 2 minutes and the supernatant was discharged. The pellet was resuspended in 978 µL of Sodium Phosphate Buffer and 122 µL of MT Buffer of the FastDNA Spin for Soil kit (MP Biomedicals, Solon, OH) and the DNA was then extracted according to the manufacturer’s instructions. Samples volume varied from 9 to 24 mL of melted ice. Further samples were collected from the bottom of nine cryoconite holes found on the Morteratsch Glacier. The DNA composition of these samples was analyzed and compared with samples from surface ice DNA extraction from the cryoconite holes samples was performed with the same kit, as for surface ice samples, from 0.7 g of sample, according to the manufacturer’s protocol.

The V4-V5 hypervariable region of the 18S rRNA gene was amplified using the eukaryotic primers 528F and 706 R^68^. A first DNA amplification was performed for each sample to evaluate its quality on the original ad on the 1:10 dilution to identify inhibition or insufficient sample. The regions were sequenced with MiSeq Illumina (Illumina, Inc., San Diego, CA) with a 2 × 250 bp paired-end protocol and Operational Taxonomic Units (OTUs) were defined with an aggregative clustering of sequences with 99% of sequence identity for the 18S rRNA gene fragment. To prepare the libraries for sequencing, a PCR was performed on the samples with GoTaq® Green Master Mix (Promega Corporation, Madison, WI) and 1 μM of each primer, for a final volume of 50 × 2 µL each. Illumina adapters (6 bp) were added at 5′ end.

The cycling conditions for the 18S rRNA fragment were: initial denaturation at 95 °C for 4 min; 30 cycles at 94 °C for 30 s, 57 °C for 30 s, and 72 °C for 30 s and a final extension at 72 °C for 5 min. Then amplicons were purified with (Wizard® SV Gel and PCR Clean-up System, Promega Corporation, Madison, WI) and quantified with Qubit® (Life Technologies, Carlsbad, CA, USA). Library preparation with the addition of standard Nextera indexes (Illumina, Inc., San Diego, CA, USA) and sequencing were carried out at Parco Tecnologico Padano (Lodi, Italy).

### DNA sequences processing and statistical analyses

The obtained reads were demultiplexed according to the indexes. Forward and reverse reads were merged only if without mismatches. Taxonomic assignation of the 18S rRNA sequences was performed by BLAST (Basic Local Alignment Search Tool) comparison against the SILVA 132 SSURef Nr 99 database^69^ assigning the sequences to the “best hit”. The ten most abundant OTUs were assigned by BLAST^70^. *A. nordenskioeldii* was the most predominant alga in all the samples, but conventional clustering methods sometimes were not able to distinguish between *A. nordenskioeldii* and *Mesotaenium* species^54^. Thus, the oligotyping pipeline^71^ was used on the *A. nordenskioeldii* sequences. Singletons (OTUs present once in one sample only) were removed from the analyses because their inclusion could inflate variance explained by multivariate analysis^72^. Analyses were performed with R 3.4.2 (R Core Team, 2014), with the VEGAN, BIODIVERISTYR, MULTTEST, MULTCOMP packages.

We normalized algal OTUs abundance to 100 to compare sequences of different samples and Hellinger-transformed them. Redundancy Analyses (RDA) were performed to see which variables significantly explained the communities variation, correcting P-values for multiple testing according to the false discovery rate (FDR) procedure^73^.

## Supplementary information

Supplementary Information.

Supplementary Information2.

Supplementary Information3.
