# Supplementary material for: Glacier algae foster ice-albedo feedback in the European Alps
Source: Sci Rep. 2020 Mar 16;10:4739. doi: 10.1038/s41598-020-61762-0 (PMC7075879; doi:10.1038/s41598-020-61762-0)
Supplement: Supplementary file 2 — Supplementary Information2. [file 41598_2020_61762_MOESM2_ESM.docx]

**Supplementary information**

**Tables:**

| Variable | df | Variance | F | P |
| --- | --- | --- | --- | --- |
| Type | 1 | 0.0491 | 16.75 | 0.001 |
| Residuals | 26 | 0.0762 |  |  |
| F_1,26_=16.75, P=0.001, Adjusted-R^2^=0.37 | | | | |

Table S2 RDA of Hellinger-transformed algal OTU abundance on type of sample based on both cryoconite and ice samples

**Figures:**


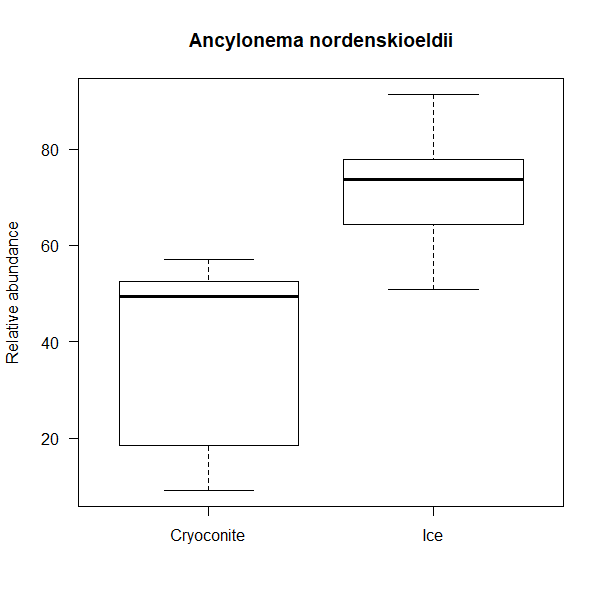


Figure S1 Boxplots that represent A. nordenskioeldii variation between ice and cryoconite samples.


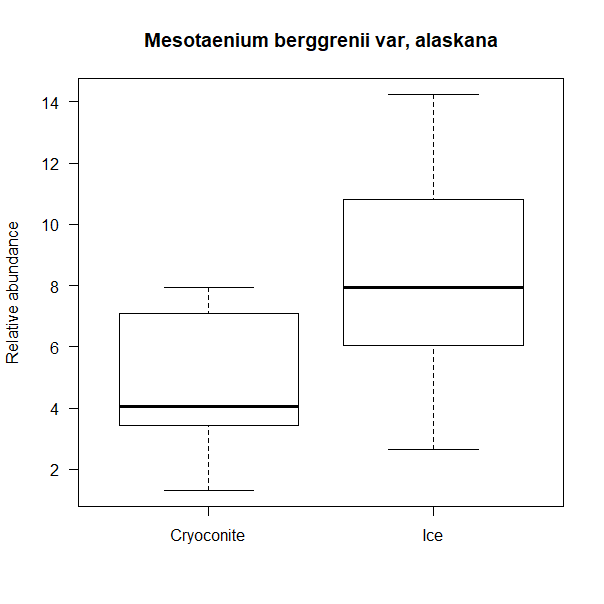


Figure S2 Boxplots that represent M. berggrenii var. alaskana variation between ice and cryoconite samples.


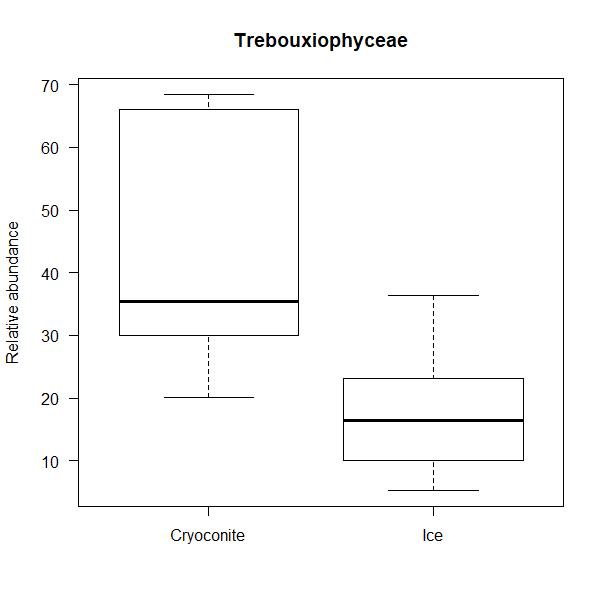


Figure S3 Boxplots that represent algae belonging to the class Trebouxiophyceae variation between ice and cryoconite samples.
